# *Chromobacterium* biopesticide overcomes insecticide resistance in malaria vector mosquitoes

**DOI:** 10.1126/sciadv.ads3658

**Published:** 2024-12-04

**Authors:** Chinmay V. Tikhe, Sare Issiaka, Yuemei Dong, Mary Kefi, Mihra Tavadia, Etienne Bilgo, Rodrigo M. Corder, John Marshall, Abdoulaye Diabate, George Dimopoulos

**Affiliations:** ^1^ W. Harry Feinstone Department of Molecular Microbiology and Immunology, Johns Hopkins Bloomberg School of Public Health, Baltimore, MD 21205, USA.; ^2^ Johns Hopkins Malaria Research Institute, Baltimore, MD 21205, USA.; ^3^ Institut de Recherche en Sciences de la Santé (IRSS) Direction Régionale de l’Ouest, Bobo-Dioulasso, BP 545, Burkina Faso.; ^4^ Université Joseph Ki-Zerbo, Ouagadougou, 03 BP 7021, Burkina Faso.; ^5^ Institut National de Santé Publique (INSP)/Centre Muraz, Bobo Dioulasso, Burkina Faso.; ^6^ School of Public Health, University of California Berkley, 2121 Berkeley Way #5328, Berkeley, CA 94720, USA.

## Abstract

Vector mosquito control is an integral part of malaria control. The global emergence of insecticide resistance in malaria-transmitting *Anophelines* has become an impediment and has created an urgent need for novel mosquito control approaches. Here, we show that a biopesticide derived from the soil-dwelling bacterium *Chromobacterium* sp. Panama (*Csp_P*) kills insecticide-resistant *Anopheles* mosquitoes, regardless of their resistance mechanisms. In addition, sublethal dose of *Csp_P* acts as a synergist to now used chemical insecticides across multiple classes. Moreover, *Csp_P* reduces host-seeking behavior and malaria parasite infection in vector mosquitoes in ways that further decrease transmission. Mosquito glutathione *S*-transferases are essential for *Csp_P*’s mosquito-killing mechanism. Enclosed field trials in Burkina Faso, conducted in diverse ecological settings and supported by a mathematical model, have now demonstrated its potential for malaria control in settings with widespread insecticide resistance.

## Teaser

*Chromobacterium* biopesticide kills insecticide-resistant malaria vector mosquitoes.

## INTRODUCTION

In 2022, there were an estimated 249 million malaria cases globally, marking an increase of 5 million from the previous year (*1*). *Anopheles* mosquitoes are the principal vectors of malaria. Vector control, primarily accomplished through chemical insecticide approaches, such as insecticide-treated bednets (ITNs) and indoor residual spraying, has contributed substantially to the reduction in malaria cases (*2*). However, widespread insecticide resistance in mosquitoes poses a challenge (*3*). It is rather difficult to find a fully insecticide-susceptible *Anopheles* population in Africa. Mosquitoes have developed multiple resistance mechanisms, including target-site mutation, metabolic resistance, high-affinity binding, cuticular resistance, and behavioral resistance (*4*, *5*). New-generation long-lasting insecticidal nets are effective only against Cytochrome P450 (CYP450)–mediated insecticide resistance (*6*). This complexity in insecticide resistance threatens progress in malaria control. Also, chemical insecticides persist in the environment and pose risks to nontarget organisms (*7*, *8*). Thus, there is a critical need for alternative, environmentally friendly mosquito control strategies that are effective against insecticide-resistant mosquitoes.

We have developed a biopesticide derived from nonlive cells of *Chromobacterium* sp. Panama (*Csp_P*) that can be delivered to mosquitoes through an artificial nectar [or attractive sugar bait (ASB)] for malaria vector control. The *Csp_P* biopesticide effectively kills *Anopheles* mosquitoes that are resistant to various insecticides, and at a sublethal dose, it restores insecticide susceptibility, making it an ideal tool for insecticide resistance mitigation and integrated vector management. Enclosed field trials in Burkina Faso have further validated the efficacy of *Csp_P* in malaria endemic conditions. In addition, *Csp_P* biopesticide inhibits host-seeking behavior and reduces mosquito permissiveness to the malaria parasite. Thus, *Csp_P* presents a bottleneck at multiple stages of malaria transmission. Laboratory studies, field trials, and modeling predictions collectively highlight the potential of the *Csp_P* biopesticide for malaria control.

## RESULTS

### The *Csp_P* biopesticide effectively kills insecticide-resistant *Anopheles* mosquitoes

**Fig. 1.**
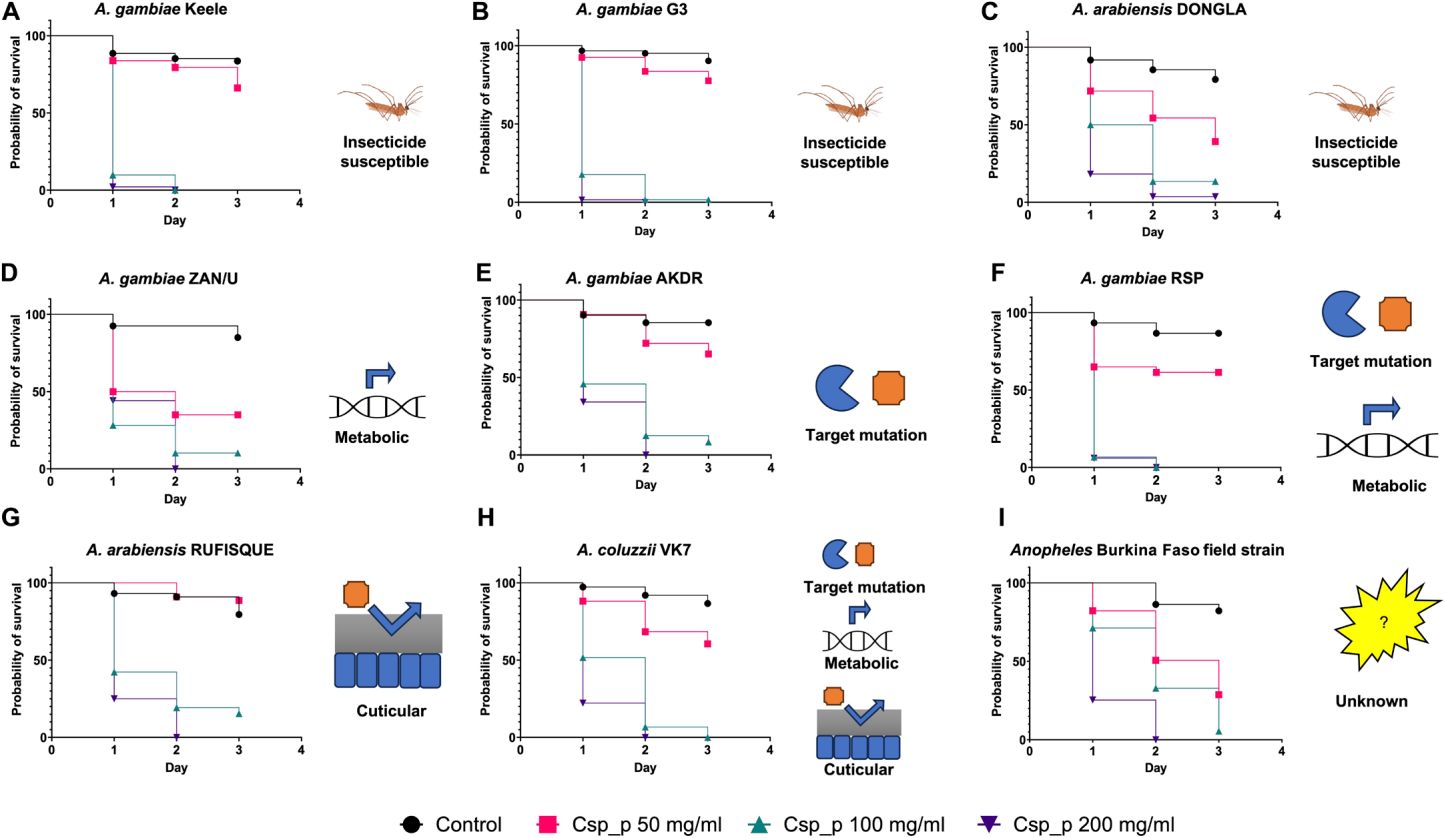
The *Csp_P* biopesticide kills insecticide-resistant *Anopheles* mosquitoes. Three-day-old adult female *Anopheles* mosquitoes fed on either a 10% sucrose solution (control) or 10% sucrose mixed with *Csp_P* bioinsecticide at concentrations of 50, 100, or 200 mg/ml for 24 hours ad libitum. Mosquito mortality was monitored over 3 days. The following *Anopheles* strains were used: insecticide-susceptible *A. gambiae* Keele (**A**) and G3 (**B**) and *A. arabiensis* DONGLA (**C**) and insecticide-resistant strains (**D**) *A. gambiae* ZAN/U (metabolic resistance), (**E**) AKDR (target site mutation), (**F**) RSP (metabolic resistance and target site mutation), (**G**) *A. arabiensis* RUFISQUE (suspected cuticular resistance), (**H**) *A. coluzzii* VK7 (target site mutation, metabolic and cuticular), and (**I**) deltamethrin-resistant field-collected *Anopheles* mosquitoes from Vallée du Kou Burkina Faso. Statistical analyses used log-rank (Mantel-Cox) test, *P* < 0.0001. The diagrams illustrating the insecticide-resistant mechanisms were created using Biorender.com.

We developed a highly potent mosquitocidal nonlive preparation of *Csp_P* (details in Materials and Methods presented in the Supplementary Materials) that is devoid of any live bacterial cells and can be readily combined with sugar solutions or artificial nectars on which mosquitoes feed [also referred to attractive toxic sugar bait (ATSB)]. The *Csp_P* biopesticide has an estimated shelf life of multiple years, as confirmed by accelerated shelf life assays (fig. S1). We assessed the efficacy of the *Csp_P* biopesticide against lab and field strains of *Anopheles* mosquitoes with various mechanisms of insecticide resistance. We used the insecticide-susceptible *Anopheles gambiae* Keele and G3 and strains; the *Anopheles arabiensis* DONGLA strain; the insecticide-resistant *A. gambiae* lab strains Akdr (target site mutation), ZAN/U (metabolic resistance *GSTe2* overexpression), and RSP (target site mutation and metabolic resistance); the *A. arabiensis* RUFISQUE (suspected cuticular resistance) strain; and the *Anopheles coluzzii* VK7 (target site, metabolic, and cuticular resistance) strain. We also used deltamethrin-resistant *Anopheles* mosquitoes collected from two field sites in Burkina Faso. Under laboratory conditions, all mosquito strains demonstrated a 100% feeding rate at all concentrations of the *Csp_P* biopesticide. Ingestion of *Csp_P* at 100 and 200 mg/ml resulted in significantly higher mortality and reduced survival probabilities for all tested mosquito lines when compared to the control [99.58% (±1.26) for *Csp_P* at 200 mg/ml and 94.10% (±6.13) for *Csp_P* at 100 mg/ml; Fig. 1]. The insecticide-resistant status or the mode of insecticide resistance did not affect the survival probability after the ingestion of *Csp_P*. However, at 50 mg/ml of *Csp_P*, a decreased but varying survival probability was observed for all strains, with an average mortality of 43.28% (±23.56), except for the *A. arabiensis* RUFISQUE strain.

### *Csp_P* insecticide synergist mechanism involves modulation of glutathione *S*-transferases

**Fig. 2.**
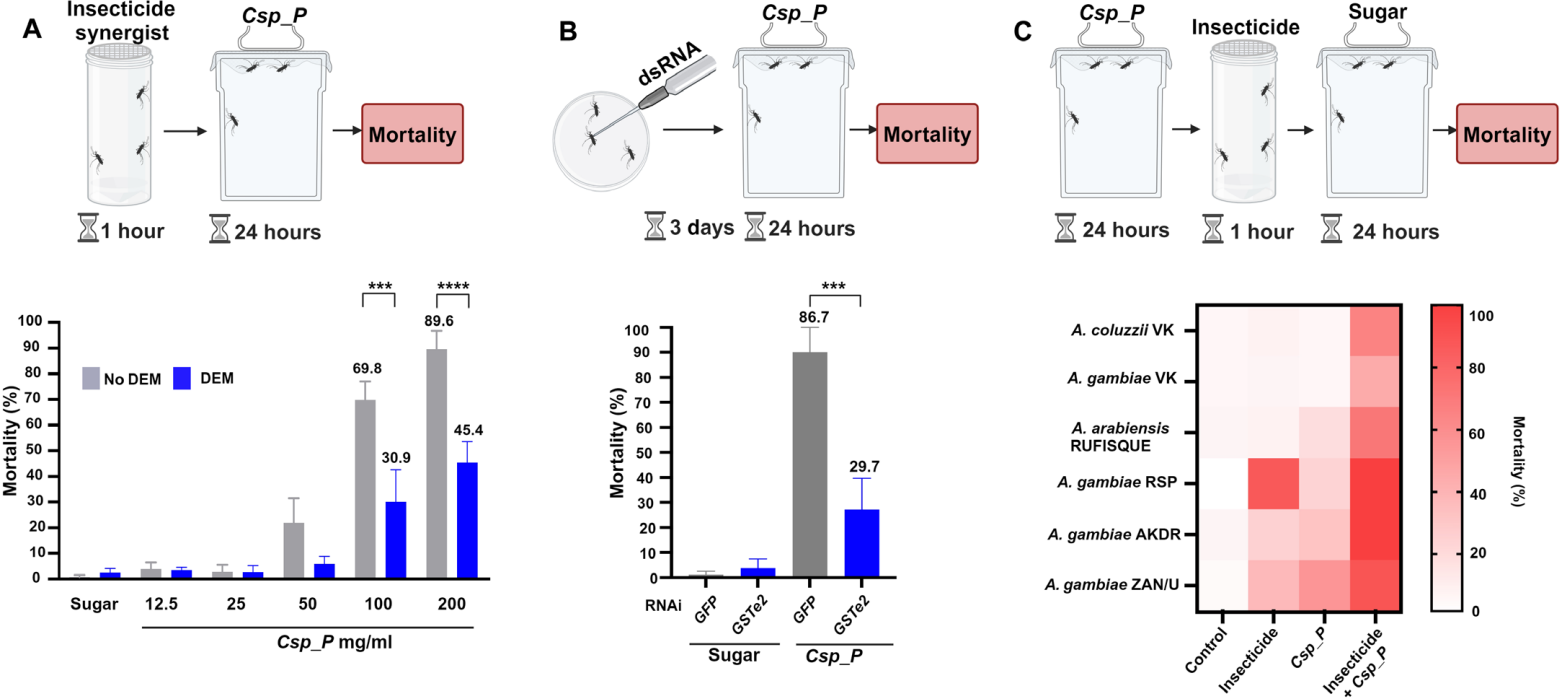
GSTs are involved in *Csp_P* -mediated toxicity, and a sublethal dose of *Csp_P* reverses insecticide resistance. (**A**) Mosquitoes preexposed to DEM postingestion of *Csp_P* (100 and 200 mg/ml) compared to mosquitoes exposed to solvent only (two-way ANOVA, followed by Šídák's multiple comparisons test, ***P = 0.002 and ****P < 0.0001; error bars represent SEM). (**B**) Mortality of *GSTe2*-silenced and control (ds*GFP*) mosquitoes post-*Csp_P* ingestion (one-way ANOVA, *P* < 0.0001, followed by Tukey’s multiple comparisons test, *P* < 0.001; error bars represent SEM). (**C**) Three-day-old adult female mosquitoes were allowed to feed on *Csp_P* (50 mg/ml) for 24 hours ad libitum. Survival of insecticide-exposed mosquitoes that had fed on *Csp_P* (50 mg/ml) for 24 hours ad libitum using the WHO tube assay. The following insecticide-resistant *Anopheles* strains and insecticide combinations were used: *A. coluzzii* and *A. gambiae* VK7 (target site mutation, metabolic and cuticular, 0.05% deltamethrin), *A. arabiensis* RUFISQUE (suspected cuticular resistance, 0.75% permethrin), *A. gambiae* RSP (metabolic resistance and target site mutation, 0.75% permethrin), AKDR (target site mutation, 0.75% permethrin), and ZAN/U (metabolic resistance, 4% DDT). Mosquitoes showed increased mortality following ingestion of *Csp_P* and subsequent exposure to chemical insecticides compared to either treatment alone (three independent replicates, 20 to 25 mosquitoes in each replicate; two-way ANOVA, *P* < 0.0001 mosquito line, *P* < 0.0001 treatment, interaction *P* < 0.0001, followed by Tukey’s multiple comparisons test; table S1). The diagrams illustrating the experiment procedures above the result panels were created using Biorender.com.

Mosquitoes protect themselves from insecticides and other xenobiotic substances through inherent detoxification mechanisms that are primarily represented by three major enzyme families: cytochrome P450 monooxygenases (CYPs), glutathione *S*-transferases (GSTs), and carboxylesterases (CCEs), while ABC transporters facilitate insecticide expulsion across cellular membranes (*4*, *9*). Our previous studies revealed that *Aedes aegypti* larvae and *A. gambiae* adults exposed to *Csp_P* exhibit altered expression of genes involved in detoxification and insecticide resistance (*10*, *11*). To gain insight into the impact of the *Csp_P* biopesticide on mosquitoes’ insecticide resistance and detoxification systems, we investigated changes in mRNA abundance of specific genes involved in insecticide metabolism and resistance. We tested the expression of four genes that are pivotal in these processes after ingestion of sublethal *Csp_P* doses and observed up-regulation of *GST*e2 and *CYP6P4*, whereas *CYP4G16* and *CYP9K1* were down-regulated (fig. S2). These findings indicate that *Csp_P* ingestion affects the detoxification machinery, influencing several genes linked to chemical insecticide resistance. To test whether these enzyme families could modulate susceptibility to *Csp_P*, we exposed mosquitoes to 4% piperonyl butoxide (PBO), 8% diethyl maleate (DEM), 10% triphenyl phosphate (TPP), and verapamil (0.01%), which are selective inhibitors of CYP, GST, CCE, and ABC transporters, respectively. Our results showed no changes in mosquito mortality following TPP or verapamil exposure post-*Csp_P* ingestion, indicating that CCE and ABC transporters are not involved in the metabolism and transport of *Csp_P* mosquitocidal metabolites (fig. S3). Preexposure to PBO resulted in increased mortality at the lowest dose (12.5 mg/ml) of *Csp_P* exposure, suggesting a protective effect of CYPs. However, PBO showed no synergy at higher doses of *Csp_P* (fig. S3). Conversely, mosquitoes exposed to DEM exhibited decreased mortality post-*Csp_P* ingestion, contrary to expectations, given the typical association of GSTs with insecticide detoxification [30.9 ± 21.65% and 45.42 ± 16.47% mortality at 100 mg/ml of *Csp_P* after preexposure to DEM, compared to mosquitoes exposed to solvent only 69.83 ± 12.53% and 89.61 ± 14.32% for *Csp_P* (100 and 200 mg/ml, respectively), one-way analysis of variance (ANOVA), *P* < 0.0001; Fig. 2A]. Silencing the *GSTe2* gene using RNA interference (RNAi) increased mosquito survival after *Csp_P* ingestion (29.67 ± 29.84% mortality in ds*GSTe2*-silenced mosquitoes post-*Csp_P* ingestion compared to *dsGFP*-injected mosquitoes at 86.67 ± 23.09%, Tukey’s multiple comparisons test, *P* < 0.005; Fig. 2B and fig. S4). At lower doses of *Csp_P* exposure, we also noted an increased mortality of the *A. gambiae* ZAN/U strain which overexpresses a mutant version of *GSTe2*, compared to other lab-maintained insecticide-resistant lines (Fig. 1) (*12*). These findings underscore the significance of GSTs, particularly GSTe2, in *Csp_P*-induced mortality. Given the interaction between the mosquito’s detoxification machinery and *Csp_P* that also involved the down-regulation of insecticide detoxification genes, we next explored whether sublethal doses of *Csp_P* could alter the mosquitoes’ susceptibility to chemical insecticides. We observed increased mortality in all insecticide-resistant *Anopheles* lines after exposure to chemical insecticides following ingestion of a sublethal *Csp_P* dose, indicating an additive synergistic effect (Fig. 2C). This synergy was independent of resistance mechanism and insecticide type. Notably, the deltamethrin-resistant *A. coluzzii* and *A. gambiae* VK7 lines exhibited increased mortality upon deltamethrin exposure post-*Csp_P* ingestion, although they only showed little synergy with PBO (fig. S5), a well-known insecticide synergist. These results highlight the potential of *Csp_P* to act as a synergist with other insecticides and to mitigate resistance.

### *Csp_P* biopesticide suppresses malaria vectors in semifield trials, and mathematical modeling predicts effective control of insecticide-resistant mosquito populations

**Fig. 3.**
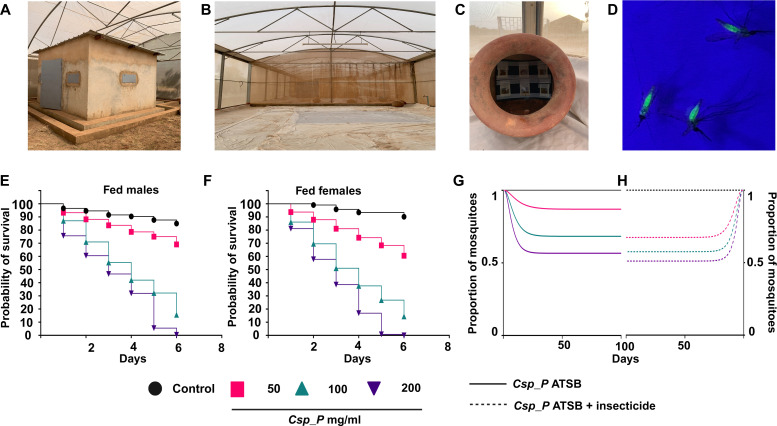
*Csp_P* demonstrates effective mosquitocidal at malaria endemic field conditions and has a predicted high efficacy in suppressing insecticide-resistant mosquito populations. Contained filed trials of *Csp_P* biopesticide were conducted in two near natural locations at Soumousoo (**A**) and Vallée du Kou (**B**) in Burkina Faso. (**C**) ATSBs were placed in clay pots to attract mosquitoes and serve as water sources. (**D**) Fluorescent dye–based screening of feeding rates of recaptured mosquitoes. Survival probabilities of males (**E**) and females (**F**) from control (50% artificial nectar) and *Csp_P* biopesticide (50, 100, and 200 mg/ml of 50% artificial nectar) exposed mosquitoes from the Vallée du Kou trials. Three independent experiments were performed with 100 female and 100 male mosquitoes in each experiment [log-rank (Mantel-Cox) test, *P* < 0.0001; fig. S9]. (**G**) Simulation model depicting mosquito survival dynamics after exposure to *Csp_p* ATSB; solid lines represent the total population of mosquitoes considering only the *Csp_p* ASTB control, or (**H**) dashed lines represent the total population of mosquitoes considering *Csp_p* ASTB plus insecticide. Simulations were performed assuming insecticide exposure rates of once every 10 days. Photo credit: Chinmay V. Tikhe, W. Harry Feinstone Department of Molecular Microbiology and Immunology, Johns Hopkins Bloomberg School of Public Health.

To further evaluate the efficacy of *Csp_P*’s mosquitocidal activity at environmental conditions found in malaria endemic environments, we conducted contained field trials with local insecticide-resistant *A. coluzzii* mosquitoes in two ecologically distinct sites in Burkina Faso. The MosquitoSphere in Soumousso emulates a typical village setting with a traditional hut, local flowering plants, and shrubs providing nectar sources, along with a small water puddle serving as a breeding site and water source for mosquitoes (Fig. 3A). The Vallée du Kou MosquitoSphere represents an open, arid area devoid of trees or huts (Fig. 3B). We used a previously developed artificial nectar, laced with a fluorescent dye to monitor feeding, that is preferred by mosquitoes over local nectar sources (Fig. 3, C and D) (*13*, *14*). Lab tests confirmed the mosquitocidal efficacy of the *Csp_P* biopesticide in this artificial nectar against *A. gambiae* Keele and *A. coluzzii* VK strains (fig. S6). In all field trials, there was no significant difference in the capture or feeding rates between male and female mosquitoes across the various *Csp_P* concentrations (fig. S7). In the Vallée du Kou trials, *Csp_P* ingestion resulted in increased mortality rates of both males and females, with the highest concentration achieving near-complete lethality (100% in females and 99.58% in males; Fig. 3, E and F). Similarly, in the Soumousso trials, *Csp_P* ingestion led to increased mortality rates in both sexes, with the highest concentration exhibiting substantial efficacy (84.66% mortality in females and 84.51% in males; fig. S8). We did not observe any difference in the survival probabilities of unfed male and female mosquitoes in any of the trials. These findings highlight *Csp_P*’s potential in combating insecticide resistance among malaria vectors in realistic and diverse ecological settings. Using a mathematical model (described in the Supplementary Materials) incorporating field-realistic feeding and insecticide exposure rates, we conducted simulations to predict the impact of *Csp_P* deployment on a mosquito population, either alone or in conjunction with ITNs (*15*). The modeling simulations indicated that deployment of *Csp_P* ATSB (200 mg/ml) alone could result in a 44% reduction of the mosquito population over time, while the presence of ITNs would lead to a total of ~50% reduction (Fig. 3, G and H). Moreover, we hypothesize that, in scenarios where mosquitoes fail to ingest sufficient biopesticide for killing, ITNs would further contribute to additional ~20% reduction in the mosquito population since *Csp_P* would act as a synergist (Fig. 3, G and H).

### *Csp_P* biopesticide modulates mosquito host-seeking behavior

**Fig. 4.**
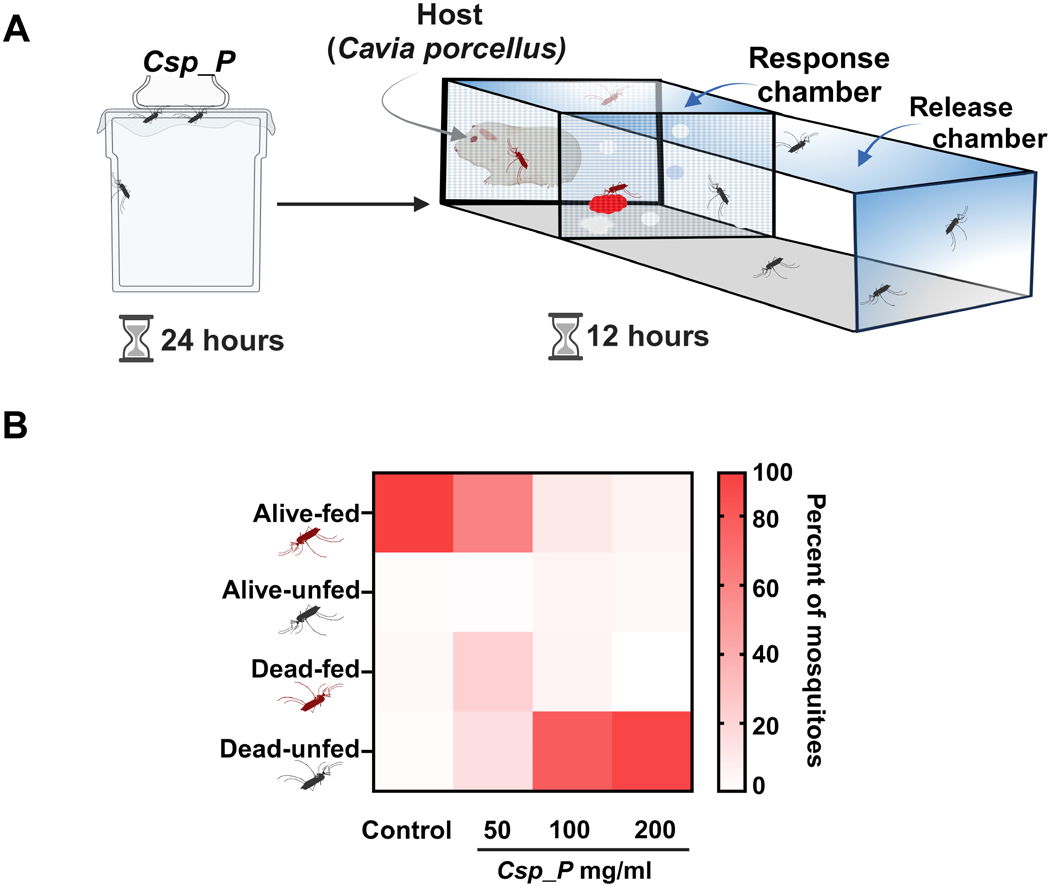
The *Csp_P* biopesticide compromises mosquitoes’ host-seeking ability. (**A**) Three-day-old adult female *A. coluzzii* VK7 (*n* = 200) mosquitoes were fed either a 5% glucose solution as a control or a 5% glucose solution laced with *Csp_P* bioinsecticide (50, 100, or 200 mg/ml) for 24 hours ad libitum. Surviving mosquitoes were subsequently placed in the release chamber. A cotton ball soaked in sugar solution mixed with red food dye was placed in the response chamber. Twelve hours later, mosquito mortality and feeding status (presence or absence of food color in the gut indicating mosquitoes’ ability to reach the response chamber) were monitored. (**B**) Mosquitoes ingesting *Csp_p* showed higher mortality and lower host seeking and feeding capability in the Tunnel assay compared to the treatment. Data derived from three independent replicates. Two-way ANOVA, followed by (Dunnett’s multiple comparisons test, *P* < 0.05, for alive-fed and dead-unfed categories, for all *Csp_P* treatments compared to the control; table S2). The diagram illustrating the experiment procedure was created using Biorender.com.

To assess the impact of *Csp_P* ingestion on mosquitoes’ host-seeking ability, we used the World Health Organization (WHO) tunnel test with female *A. coluzzii* mosquitoes. Insecticide-resistant mosquitoes were released into the chamber and observed for their capacity to navigate through a holed netting to reach the response chamber, which is near a bait animal serving as the host (Fig. 4). The mosquitoes displayed an inability to respond to host stimuli across all concentrations of *Csp_P* evaluated. This effect was particularly pronounced at the two highest concentrations of 100 and 200 mg/ml, where a majority of the mosquitoes died within the tunnel without ever reaching the response chamber (77% at 100 mg/ml and 89% at 200 mg/ml; Fig 4). This assay shows that *Csp_P* ingestion abolishes mosquitos’ ability to respond to host stimuli, effectively hindering host seeking and blood feeding, which are essential for malaria transmission.

### *Csp_P* biopesticide decreases mosquito vector competence

**Fig. 5.**
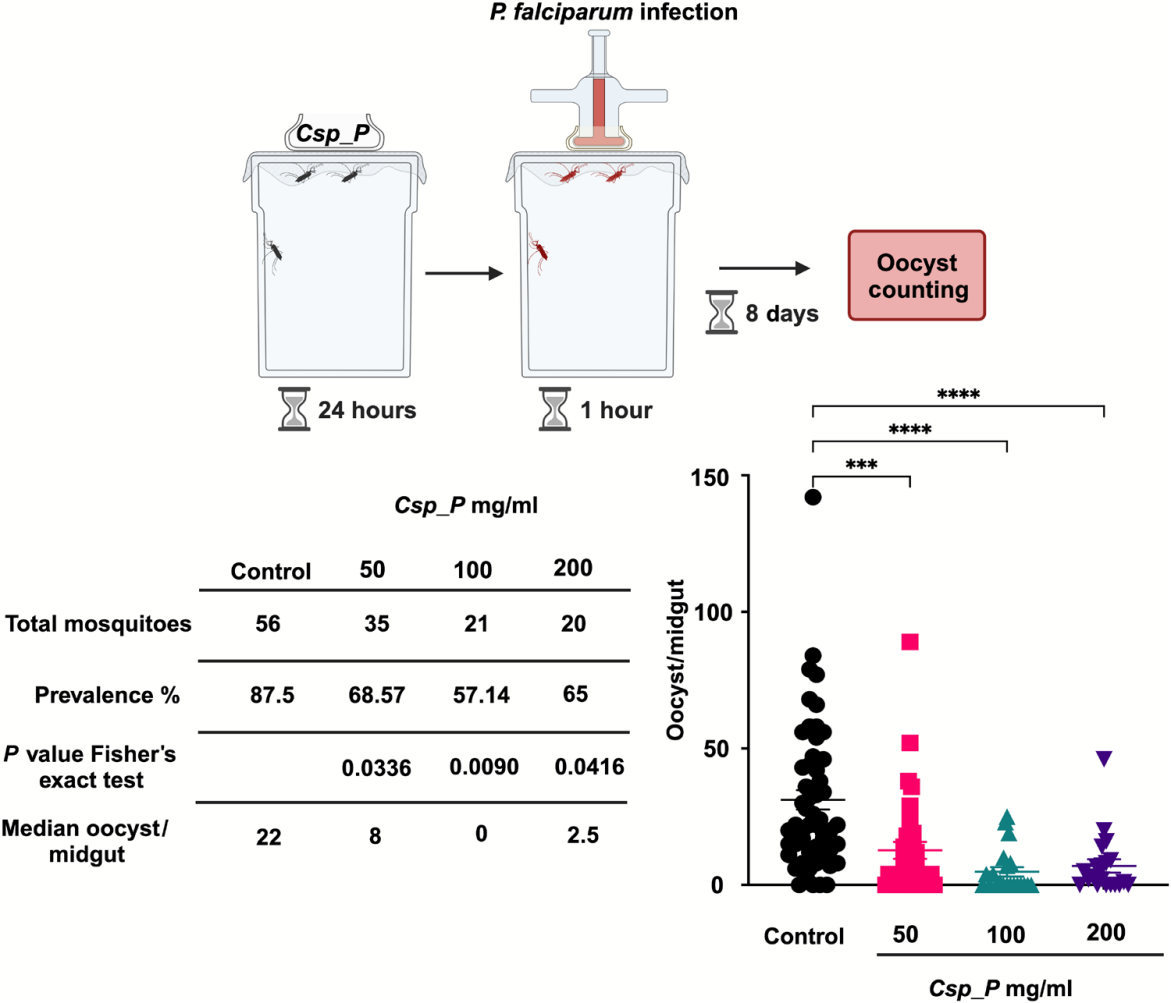
The *Csp_P* biopesticide renders *A. gambiae* Keele mosquitoes more resistant to human malaria parasite infection. Four treatment groups comprised mosquitoes fed on 10% sucrose alone (control) or laced with *Csp_P* bioinsecticide (50, 100, or 200 mg/ml) for 24 hours ad libitum. Surviving mosquitoes were then fed with *P. falciparum* gametocyte culture mixed blood, and oocysts were counted at 8 days postinfection. Data were pooled from four independent replicates, with each data point representing the number of oocysts in an individual mosquito and the red line indicating the median number (Kruskal-Wallis test, followed by Dunn’s multiple comparisons test, ***P < 0.001 and ****P < 0.0001). Inset table provides total mosquitoes (*N*), infection prevalence (% of mosquitoes infected with at least one oocyst), and Fisher’s exact test *P* value for infection prevalence. The top illustration was created using Biorender.com.

Next, we tested whether ingestion of *Csp_P* influenced mosquito susceptibility, or vector competence, to the human malaria parasite *Plasmodium falciparum*. Mosquitoes were allowed to feed on *Csp_P*, and the surviving mosquitoes were subsequently fed on *P. falciparum*–infected human blood through a membrane feeder (Fig. 5). Similar to the findings from the tunnel test, the majority of mosquitoes that fed on *Csp_P* either did not feed on *Plasmodium*-infected blood or did not survive until day 8 postinfection, the point at which the infection phenotype is normally determined. However, the few *Csp_P* preexposed mosquitoes that fed on the infected blood and succeeded in surviving to day 8 exhibited significantly lower oocyst numbers than did the control group that had not ingested the *Csp_P* biopesticide (Fig. 5). These results show that even if mosquitoes succeed to blood-feed on malaria-infected hosts after *Csp_P* ingestion, they are less likely to transmit the parasite due to decreased vector competence.

## DISCUSSION

The great majority of malaria cases occur in sub-Saharan Africa, imposing a major socioeconomic and public health burden (*1*). Given the lack of efficient vaccines and the emergence of drug-resistant *Plasmodium* parasites, controlling vector mosquito populations remains among the most effective strategies for malaria control. However, because of the ongoing emergence of mosquito insecticide resistance, conventional vector control is in dire need of reexamination and revision (*3*). Microbial biopesticides, most commonly composed of nonlive crude bacterial cell lysates, offer an eco-friendly alternative to chemical insecticides with multiple advantages (*16*). As these insecticides consist of dead cells, there is no risk of environmental spread and pathogenicity, and they typically show minimal environmental toxicity due to rapid degradation. Their complex nature, frequently comprising multiple insecticidal molecules with diverse target specificities, makes developing resistance difficult (*10*). The public acceptance and the regulatory environment of microbial biopesticides are also more favorable than their chemical counterparts. The Insecticide Resistance Action Committee has recognized the potential of novel microbial biopesticides and classified them into a unique category: bacterial insecticides with an unknown mode of action (UNB) (*17*). As of today, a robust biopesticide that can be used for the control of adult mosquitoes does not exist.

*Csp_P* is a soil and water associated nonspore-forming Gram-negative bacillus belonging to the genus *Chromobacterium* (*18*). Live *Csp_P* is highly pathogenic for *Aedes*, *Anopheles*, and *Culex* mosquitoes, and a nonlive preparation of *Csp_P* dried biofilm efficiently kills *Aedes* and *Anopheles* larvae in the lab and field (*19*). Exposure to sublethal doses of the *Csp_P* biopesticide over 10 continuous generations at the larval stage does not result in the development of resistance in mosquito populations (*10*), and a crude biofilm extract of *Csp_P* does not show cytotoxic effects on mammalian cells (*18*). Because of its flexible scalability, *Csp_P* can be cost-effectively produced locally, with minimal training, in malaria-endemic countries. Given these attributes, the *Csp_P* biopesticide has a promising potential for the control of insecticide-resistant *Anopheles* mosquitoes.

Feeding on sugar-rich substrates such as flower nectars is an essential part of mosquito biology that can be exploited to expose the insect to insecticides using artificial nectars. Accordingly, ATSB is an innovative way of delivering insecticides to their target with minimal environmental exposure and off-target effects (*20*). ATSBs can be explicitly formulated to attract target insects and can even be designed to deter feeding by nontarget insects. The *Csp_P* biopesticide can be easily incorporated into attractive artificial nectar to be ingested by mosquitoes. Although ATSB technology has been explored for mosquito control for the past few decades, its efficacy on a broader scale has not yet been evaluated. Recently, however, field trials have shown success with ATSBs infused with chemical insecticides, and the technology continues to evolve and develop (*13*, *21*).

Lab tests showed that the *Csp_P* biopesticide effectively kills adult mosquitoes of multiple *Anopheles* lab strains with varying mechanisms of insecticide resistance, and it also kills field-collected insecticide-resistant mosquitoes. *Csp_P*-mediated mortality was not found to be dependent on the mechanism of insecticide resistance, indicating that the mode of action of *Csp_P* is distinct from that of the now used insecticides and that it can potentially be used to control all insecticide-resistant mosquitoes.

To gain insights into possible interactions between *Csp_P* and the mosquito’s insecticide and xenobiotic detoxification systems, we used selective inhibitors of enzyme families that play key roles in such processes upon *Csp_P* exposure. Preexposure of mosquitoes to the CYP450 inhibitor, PBO, increased their mortality postingestion of otherwise sublethal doses of *Csp_P*, indicating that CYP450s, to a certain extent, protect mosquitoes from *Csp_P*. Unexpectedly, inhibition or suppression of GST activity by either exposure to its inhibitor DEM or by RNAi-mediated *GST* gene silencing resulted in a higher mosquito survival post-*Csp_P* ingestion. Conversely, we observed increased mortality of the *GSTe2*-overexpressing line *A. gambiae* ZAN/U upon *Csp_P* ingestion (*12*). *GSTe2*-mediated insecticide resistance of *Anopheles* is well documented, and GSTs are generally known for their role in detoxification processes, specifically in phase 2 through either a direct mechanism involving insecticide metabolism and sequestration or indirectly by protecting against insecticide-mediated oxidative stress (*12*). However, although GSTs’ enzymatic function generally results in less reactive metabolites, several cases in which the GSTs form toxic compounds have been reported. For example, GSTs have been linked to the formation of a cisplatin conjugate that is more nephrotoxic than the anticancer drug itself (*22*), and GSTs have also been implicated in the bioactivation of haloalkanes and haloalkenes, resulting in the formation of highly cytotoxic reactive intermediates (*23*). Our data indicate that mosquito’s GSTs play a role in *Csp_P* biopesticide–mediated toxicity, likely through the production of toxic conjugates with compounds present in the *Csp_P* biopesticide.

Ingestion of a sublethal dose of *Csp_P* renders insecticide-resistant mosquitoes susceptible to chemical insecticides to which they were genetically resistant. The mechanism of this synergistic effect is not clear but could involve insecticide detoxification genes that were down-regulated upon *Csp_P* exposure. This synergistic effect of *Csp_P* is independent of the insecticide resistance mechanism and is highly important from a mosquito control perspective. While it is quite possible that some mosquitoes may not ingest a sufficient amount of *Csp_P* from the ATSB to kill them, even a sublethal amount will make them more susceptible to now used insecticides. This dual mode of action, being mosquitocidal at high doses and functioning as an insecticide synergist at low doses, makes *Csp_P* biopesticide a potentially ideal tool for the control of insecticide-resistant mosquitoes. Mathematical modeling with lab and field data predicts a 40 to 50% reduction in a fully insecticide-resistant mosquito population after integrating *Csp_P* into current vector management programs. It is also noteworthy that we have previously shown that continuous exposure of mosquitoes to sublethal doses of *Csp_P* for 10 generations does not result in the development of resistance to *Csp_P* or cross-resistance to other chemical insecticides (*10*).

Acquisition of blood meals from human hosts is essential for malaria transmission. We show that *Csp_P* hampered the mosquitoes’ ability to host-seek, and this biopesticide thereby imposes an additional barrier to malaria transmission. *Csp_P* ingestion also renders the mosquito less permissive to *P. falciparum* infection, thereby imposing a third transmission barrier. Inhibition of *Plasmodium* is most likely mediated by the *Csp_P* -produced histone deacetylase inhibitor, romidepsin (*24*).

In summary, we show that the *Csp_P* biopesticide kills insecticide-resistant *Anopheles* mosquitoes, regardless of resistance mechanism, and, at sublethal doses, induces susceptibility to chemical insecticides along with inhibition of host seeking and parasite infection. The environmentally friendly *Csp_P* biopesticide thereby imposes multiple bottlenecks at critical junctions of the malaria transmission cycle and may therefore qualify as a powerful weapon against malaria.

## MATERIALS AND METHODS

### Ethics statement

This study was conducted according to the recommendations of the Guide for the Care and Use of Laboratory Animals of the National Institutes of Health, the Animal Care and Use Committee of Johns Hopkins University, and the Institutional Ethics Committee. The Institutional Animal Care and Use Committee approved the protocol number MO21H10. Mice were used for rearing mosquitoes. Anonymous, commercial blood from human donors was used for *P. falciparum* gametocyte cultures and infection assays in mosquitoes.

### General experimental design and statistics

All the experiments were carried out at least three times independently unless otherwise stated. Data were analyzed using appropriate statistical tests in GraphPad Prism version 10.2.3 for Windows. The corresponding tests used are described in the figure legend.

### Lab-based preparation of *Csp_P* biopesticide

A frozen glycerol stock of *Csp_P* was streaked on a Luria-Bertani (LB) agar plate to obtain individual colonies. A single colony of *Csp_P* was inoculated into 10 ml of fresh yeast extract broth [YEB; 10 g of tryptone, 5 g of yeast extract, 5 g of sucrose, 5 g of sodium chloride, and 0.25 g of MgSO_4_*7H_2_O in 1000 ml of deionized (DI) H_2_O; Sigma-Aldrich] in a 50-ml falcon tube and cultured overnight at 30°C with shaking at 250 rpm. This overnight-grown seed culture was then inoculated into 1 liter of YEB in a 2-liter glass flask and incubated at 30°C with agitation at 250 rpm for 72 hours, followed by 72 hours of stationary incubation with oxygen deprivation. The culture was then transferred to sterile 22 cm by 22 cm by 2 cm petri dishes (Thermo Fisher Scientific) and incubated at room temperature for 7 days to promote biofilm formation. Afterward, the *Csp_P* culture was transferred to silicone trays (dimension: 27 cm by 30 cm by 1 cm; Amazon #B087NGPP99) and dried in a food dryer at 70°C. The dried biomass, along with spent fermentation media, was ground into a uniform, fine powder in a food blender. The dried *Csp_P* powder was diluted with DI water and spread on LB agar (Sigma-Aldrich) plates or inoculated into 5 ml of LB broth to verify the absence of live bacterial cells in the preparation. Microscopic examination at ×1000 magnification was performed to ensure no intact bacterial cells remained in the preparation.

### Attractive bait

For all the experiments, a proprietary artificial nectar (ASB) provided by Westham Co. (https://westhamco.com/) was used. Unless specified otherwise, the artificial nectar was diluted with sterile distilled water to a final concentration of 50%.

### Mosquito rearing

*A. gambiae* Keele strain was reared in the insectary at the Johns Hopkins Malaria Research Institute, Baltimore, MD, USA, at 27°C and 80% relative humidity with a 14:10-hour light-dark cycle. Live eggs of the following strains were obtained from MR4 through Biodefense and Emerging Infections Research Resources Repository (BEI) Resources: *A. gambiae* insecticide–susceptible strain G3 (MRA-112); *A. arabiensis* insecticide–susceptible strain DONGLA (MRA-856); *A. gambiae* insecticide–resistant strains RSP (MRA-334), AKDR (MRA-1280), and ZAN/U (MRA-594); and *A. arabiensis* insecticide–resistant strain RUFISQUE (not available for ordering). All the strains mentioned above were reared in the Johns Hopkins Malaria Research Institute, Baltimore, MD, USA, at 27°C and 80% relative humidity with a 14:10-hour light-dark cycle. A new batch of eggs was used for each replicate. *A. gambiae* VK7 (insecticide resistant), *A. coluzzii* VK7 (insecticide resistant), and *A. gambiae* Kisumu (insecticide susceptible) were reared in the insectary at the Institut de Recherche en Sciences de la Santé (IRSS), Bobo Dioulasso, Burkina Faso, at 27°C and 80% relative humidity with a 14:10-hour light-dark cycle. *A. gambiae* and *A. coluzzii* strains are maintained as separate colonies at IRSS Burkina Faso and are routinely validated with polymerase chain reaction to avoid cross-contamination.

### Field collection of mosquitoes

*Anopheles* larvae were collected from rice fields in Soumousso and Vallée du kou in Burkina Faso, respectively. Larvae were brought to the insectary and reared to adults by feeding on fish meal. Female mosquitoes were used to test the efficiency of *Csp_P*.

### Bioassay of adult mosquitocidal activity of *Csp_P* biopesticide in the lab

*Csp_P* biopesticide was mixed and thoroughly dissolved in either 10% sucrose, 5% glucose, or 50% artificial nectar containing 0.01% fluorescein to obtain the final concentrations of 50, 100, and 200 mg/ml. These concentrations were evaluated against lab and field-collected mosquito strains mentioned above in a cup bioassay. Briefly, 700 μl of *Csp_P* biopesticide in either 10% sucrose, 5% glucose, or artificial nectar with Ffuorescein (0.01%) was added to the lids of 5-ml polystyrene round-bottom tubes (Falcon #352054). Lids were sealed with stretched parafilm, similar to the mounted parafilm attached to an artificial glass feeder used for blood feeding and perforated with an insulin syringe needle. A total of 20 female mosquitoes, starved for 24 hours with access only to water (provided with water-soaked cotton balls), were introduced into 8-oz (236.588 ml) paper cups. Mosquitoes were allowed to feed on *Csp_P* solution ad libitum for 24 hours. Dead and live mosquitoes were screened for the presence of fluorescein, indicating their feeding status. After the 24-hour feeding period, surviving mosquitoes were provided with 10% sucrose water, and mortality was monitored over the next 2 days.

### Accelerated shelf life testing of *Csp_P* biopesticide

According to the Environmental Protection Agency (EPA) guidelines, accelerated shelf life tests were performed to determine the stability of *Csp_P* biopesticide. *Csp_P* biopesticide dry powder was incubated in a glass petri dish at 54°C for 2 weeks, equivalent to 1 year of incubation at room temperature (EPA guideline OPP: 830.6317). *Csp_P* biopesticide was also subjected to 70°C incubation for 2 weeks. In parallel, *Csp_P* biopesticide was incubated at room temperature for 2 weeks as a control. After incubation at respective temperatures, *Csp_P* biopesticide was fed to *A. gambiae* Keele females by mixing with the artificial nectar as described above.

### Insecticide susceptibility and synergist assays

Insecticide susceptibility assays were carried out according to the WHO bioassay protocol using insecticide-treated papers. *A. gambiae* insecticide–resistant strains RSP, AKDR, ZAN/U, *A. arabiensis* RUFISQUE, *A. gambiae* VK7, and *A. coluzzii* VK7 were starved for 24 hours and fed on either 10% sucrose or *Csp_P* (50 mg/ml) in 10% sucrose ad libitum. Surviving mosquitoes that were actively flying and found on the net and the upper wall of the cup were used for the insecticide exposure assays. Mosquitoes fed on sugar and *Csp_P* were exposed to either the control solvent or solvent with an insecticide at the WHO-recommended discriminatory concentration for 1 hour. Mortality was measured 24 hours after insecticide exposure. A minimum of 15 females were used for each treatment. For RSP, AKDR, and RUFISQUE, 0.75% permethrin papers was used; for ZAN/U, 4% DDT papers was used; and for *A. gambiae* VK7 and *A. coluzzii* VK7, 0.05% deltamethrin papers was used. All the insecticide papers, along with their respective controls, were ordered from the Vector Control Research Unit, School of Biological Sciences, Universiti Sains Malaysia.

For synergist exposure assays, grade 1 12 cm by 15 cm Whatman papers were impregnated with either 4% PBO, 8% DEM, or 10% TPP in Dow Corning 556 cosmetic grade fluid and acetone. For verapamil assays, Whatman papers were impregnated with 0.01% verapamil in methanol and Dow Corning 556 cosmetic grade fluid. Papers impregnated with solvents alone were used as controls. Four- to 5-day-old *A. gambiae* Keele females were preexposed to PBO, DEM, TPP, or verapamil for 1 hour, followed by feeding on *Csp_P* doses ranging from 12.5 to 200 mg/ml in 10% sucrose. Sucrose (10%) alone was used as a control. Live and dead mosquitoes were counted 24 hours later.

### Gene expression analysis through quantitative reverse transcription polymerase chain reaction and RNAi-mediated gene silencing assays

Three-day-old *A. gambiae* Keele female mosquitoes were starved for 24 hours and fed on *Csp_P* (50 mg/ml) ad libitum for 24 hours. RNA was extracted from a pool of five surviving whole mosquitoes using the TRIzol (Thermo Fisher Scientific) extraction protocol according to the manufacturer’s instructions. Gene expression of selected detoxification genes was measured using a previously described protocol with primers described in table S1 (*25*–*27*). *A. gambiae* (RPS7) housekeeping gene was used for normalization, and the fold change of each gene was calculated using the ΔΔ*Ct* method (*27*).

RNAi-mediated gene silencing of *GSTe2* (AGAP009194) was performed according to a previously described protocol with primers listed in table S1 (*26*). The silencing efficiency of *GSTe2* was confirmed 3 days postinjection of *dsRNA* by comparing *GSTe2* expression in mosquitoes injected with control *GFP dsRNA*.

To assess the effect of *GSTe2* silencing on *Csp_P*-mediated mortality, *Gste2* gene–silenced or *GFP dsRNA*–injected control mosquitoes were fed on either 10% sucrose or *Csp_P* biopesticide (25 mg/ml) for 25 hours ad libitum. Mortality rates were then recorded 24 hours later. It is worth noting that, at all concentrations of *Csp_P* exceeding 25 mg/ml, both the control *GFP dsRNA* and *GSTe2 dsRNA*–injected mosquitoes exhibited 100% mortality. This outcome may be attributed to physiological changes or a stress response triggered by the injection-related injury. Therefore, *Csp_P* (25 mg/ml) was selected as the concentration for this experiment.

### Preparation of feeding stations for semifield testing

Individual pouches were assembled by sealing a piece of 2″ by 2″ parafilm to a 2″ by 3″ Ziploc bag using a heat sealer along three sides, leaving the top side open. Each pouch was loaded from the top with 2 ml of artificial nectar containing 0.01% fluorescein, serving as the control, or the mixtures of *Csp_P* at concentrations of 50, 100, or 200 mg/ml, as specified in the respective experiments. The open side was then sealed with sticky autoclave tape. To allow mosquitoes access to the sugar bait, approximately 10 holes were punctured through the parafilm side of each pouch using a 30G insulin syringe. Six pouches were evenly attached on a black Styrofoam sheet (9″ by 12″) in two rows using sticky autoclave tape.

### Near-natural semifield testing

Two independent semifield trials of *Csp_P* biopesticide were conducted at two near-natural environmental but ecologically distinct sites in Burkina Faso. The first trial took place at the MosquitoSphere in Soumousso, characterized by a typical village-like environment in many West African countries surrounded by large trees. This MosquitoSphere facility comprises six individual compartments, each featuring a traditional West African WHO hut, local plants and shrubs, and a small water puddle, previously used for other similar field trials. Here, two feeding stations were hung outside the hut, and two stations were placed inside the hut. Within the compartment, stations were set up with either control bait (artificial nectar, 0.01% fluorescein) or bait containing *Csp_P* at concentrations of 50, 100, or 200 mg/ml. The trials involved deltamethrin-resistant *A. coluzzii* VK7 strain, released at 18:00 hours as 3-day-old, 4-hour- starved batches of 100 male and 100 female mosquitoes. After 12 hours, dead mosquitoes were collected in individual plastic tubes, while live mosquitoes were captured with mouth aspirators, transferred to large paper cups, and transported to the lab. Live mosquitoes were knocked down on ice and sorted under ultraviolet light to determine feeding status. Mosquitoes were then separated according to sex and feeding status, placed in large paper cups, and provided with a 5% glucose solution. Mosquito mortality was monitored daily for 6 days. Chambers were shuffled between replicates to ensure different treatments in each compartment to eliminate positional biases.

The second field trials were conducted at the Vallée du kou MosquitoSphere, which is designed to represent an open, arid ecological setting devoid of trees, shrubs, water puddles, and huts. The experimental setup mirrored that of Soumousso.

### Tunnel test

The tunnel test chamber consists of two compartments: a release chamber measuring 40 cm by 25 cm by 25 cm and a response chamber measuring 20 cm by 25 cm by 25 cm. These two chambers are separated by a net with holes large enough to allow the mosquitoes to fly through. The end of the response chamber is netted, and a bait animal is placed just outside this netting. A total of 200 female mosquitoes from *A. gambiae* VK7 and *A. coluzzii* VK7 strains, aged 3 days and starved for 24 hours, were fed ad libitum on either 5% glucose or 5% glucose mixed with *Csp_P* at concentrations of 50, 100, or 200 mg/ml for 24 hours. Surviving mosquitoes were then released into the release chamber. In the response chamber, a cotton ball soaked in 5% glucose with 1% red food dye was placed as a food resource. An immobilized guinea pig (*Cavia porcellus*) was positioned outside the response chamber to act as bait. After 12 hours, dead and live mosquitoes were counted and categorized as fed or unfed based on the presence of red food dye in their guts.

### Mathematical modeling

To characterize the dynamics of mosquitoes exposed to ATSBs, we used a previously published model (*15*) to describe the dynamics of malaria vector control following ATSB exposure. The modeling approach considers species-specific sugar-feeding rates, the absence of dye decay, and a constant rate of mosquito emergence. The system of equations represents the framework

$$\begin{array}{c} \frac{\mathrm{dU}}{\mathrm{dt}}=\mathrm{bN}-\left(s+\mu \right)U\\ \frac{\mathrm{dM}}{\mathrm{dt}}=\mathrm{sU}-\mu_{\text{ATSB}}M \end{array}$$ (1)

Here, U and M represent the density of unfed and fed mosquitoes, respectively. Parameters include b for a constant adult emergence rate, adjusted to match the death rate μ, so that the population is at equilibrium in the absence of ATSB. Unfed mosquitos represent those not yet exposed to the ASTB. They may eventually be exposed (i.e., fed) according to the sugar-feeding rate s. Following exposure to ATSB, mosquitoes are subjected to a death rate $\mu_{\text{ATSB}}$ (with $\mu_{\text{ATSB}}>\mu$).

We modified the model represented by Eq. 1 (*15*) and introduced a new variable I to additionally account for insecticide exposure. Since all mosquitoes are insecticide resistant and are only affected by insecticide after being exposed to ATSB, we assume that only fed mosquitoes (those in the compartment M after exposure to ATSB) move to the compartment I according to the rate of insecticide exposure i. Mosquitoes in compartment I are susceptible to insecticide and subjected to a mortality rate $\mu_{\text{ATSB}+i}$ (with $\mu_{\text{ATSB}+i}>\mu_{\text{ATSB}}$). The modified model is represented by the equations

$$\begin{array}{c} \frac{\mathrm{dU}}{\mathrm{dt}}=\mathrm{bN}-\left(s+\mu \right)U\\ \frac{\mathrm{dM}}{\mathrm{dt}}=\mathrm{sU}-\mu_{\text{ATSB}}M-\mathrm{iI}\\ \frac{\mathrm{dI}}{\mathrm{dt}}=\mathrm{iI}-\mu_{\text{ATSB}+i}I \end{array}$$ (2)

where I represents the density of mosquitoes exposed to insecticide after ATSB exposure. The modified modeling framework is diagrammatically represented in fig. S10.

The mosquito baseline mortality rate was assumed to be 0.094 ${\text{day}}^{-1}$ (*15*). We used Cox proportional hazards models to fit experimental data of different CSP concentrations and determine the mosquito species–specific mortality ratio after exposure to ATSB. The *Anopheles coluzzii* species–specific mortality ratio ($\mu_{\text{ATSB}}/\mu$) for different *Csp_P* concentrations obtained from the Cox analysis (with 90% confidence intervals in parentheses) were 2.53 (1.95 to 3.30) for *Csp_p* (50 mg/ml), 7.71 (6.07 to 9.79) for *Csp_p* (100 mg/ml), and 13.00 (10.26 to 16.48) for *Csp_p* (200 mg/ml).

Regarding the insecticide exposure rates i, we simulated three different scenarios in which mosquitoes are exposed to the insecticide, on average, once every 10, 5, or 2 days. In all simulations, we maintained a constant sugar-feeding rate $s=0.15\;{\text{day}}^{-1}$ (*15*). For simplicity, we assume that mosquitoes exposed to the insecticide after ATSB exposure have a mortality rate twice as high as those only exposed to ATSB.

### *P. falciparum* infection assays

Following ingestion of *Csp_P*, surviving mosquitoes were subsequently fed on *P. falciparum* NF54 gametocyte cultures (from MR4) through artificial membrane glass feeders as described (*28*). Mosquitoes fed on the nectar were used as controls. After removing unfed females, mosquitoes were kept for 8 days at 27°C before their midguts were dissected in phosphate-buffered saline and stained with 0.1% mercurochrome to count oocysts. Each experiment included at least four independent biological replicates, with a minimum of 50 mosquitoes per replicate. Statistical analysis included the Mann-Whitney test to compare infection intensities between control and treated groups, while Fisher’s exact test determined the significance of infection prevalence (percentage of mosquitoes with at least one oocyst). Graphs presenting oocyst counts (dot plots with median values) were generated using GraphPad Prism 10 software.
