# Supplementary material for: Chromobacterium biopesticide overcomes insecticide resistance in malaria vector mosquitoes
Source: Sci Adv. 2024 Dec 4;10(49):eads3658. doi: 10.1126/sciadv.ads3658 (PMC11616681; doi:10.1126/sciadv.ads3658)
Supplement: Supplementary file 1 — Figs. S1 to S10 Tables S1 to S3 [file sciadv.ads3658_sm.pdf]

Science Advances
AAAS

Supplementary Materials for

Chromobacterium biopesticide overcomes insecticide resistance in malaria vector mosquitoes

Chinmay V. Tikhe *et al.*

Corresponding author: George Dimopoulos, gdimopo1@jhu.edu

Sci. Adv. **10**, eads3658 (2024)
DOI: 10.1126/sciadv.ads3658

This PDF file includes:

Figs. S1 to S10
Tables S1 to S3

Supplementary Figures

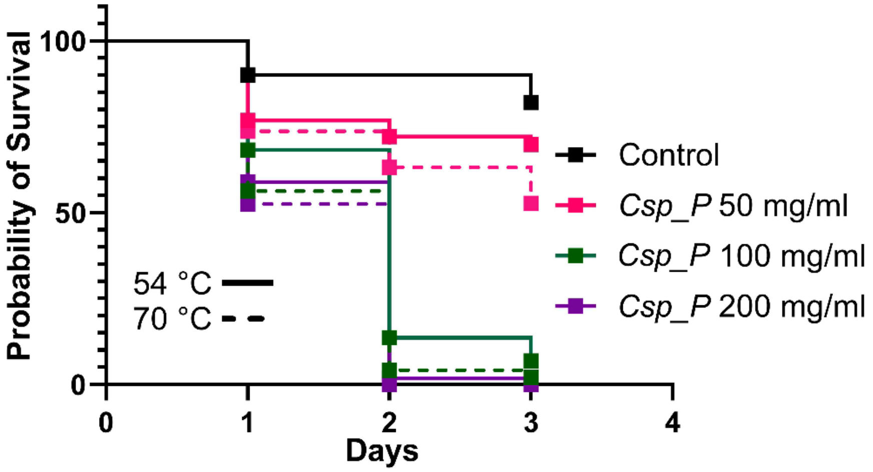

Fig. S1. Accelerated shelf-life testing of *Chromobacterium* sp. Panama (*Csp_P*) biopesticide

Csp_P powder was incubated at either 54°C or 70°C for two weeks. The powder was mixed with 10% sucrose at concentrations of 50, 100, or 200 mg/ml; 10% sucrose solution was used as a control. 3–5-day old *An. gambiae* Keele female mosquitoes were starved for 24 hours and were allowed to feed on either *Csp_P* biopesticide or control (10% sucrose) for 24 hours ad-libitum. There was a significant decrease in survival probabilities of mosquitoes fed on all the concentrations of *Csp_P* compared to the control (Log-rank (Mantel-Cox) test, $p < 0.0001$). Incubation at either 54°C or 70°C for two weeks did not result in loss of mosquitocidal activity of *Csp_P* biopesticide. Each replicate included at least 20 mosquitoes, and a total of three biological replicates were conducted.

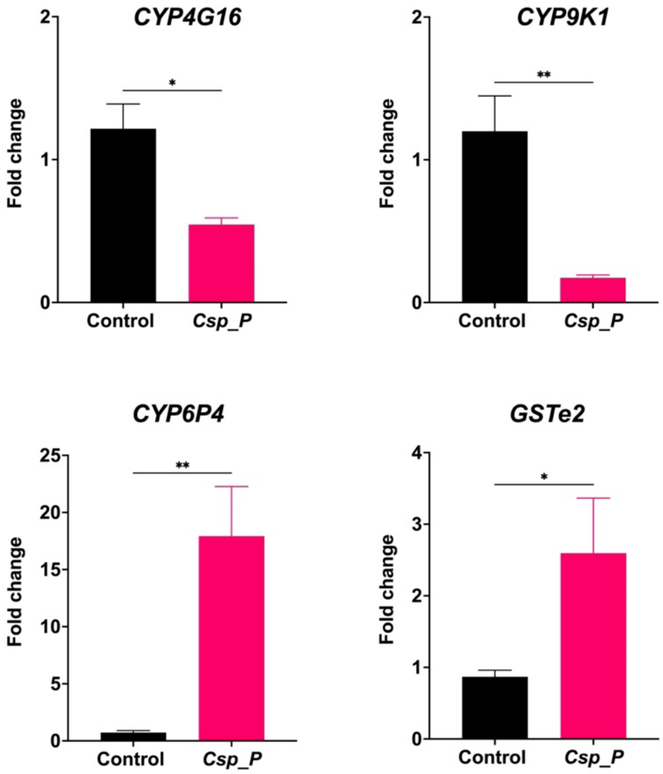

Fig. S2. Changes of gene expression of selected detoxification genes following ingestion of *Chromobacterium* sp. Panama (*Csp_P*) biopesticide

3–5-day old *An. gambiae* Keele female mosquitoes were starved for 24 hours and then allowed to feed on either *Csp_P* biopesticide (50 mg/ml) or control (10% sucrose) for 24 hours ad-libitum. Gene expression changes in the *CYP4G16*, *CYP9K1*, *CYP6P4,* and *GSTe2* were analyzed in whole mosquitoes using qRT-PCR with gene-specific primers. *CYP4G16*, *CYP9K1* were down regulated while *CYP6P4* and *GSTe2* were upregulated post *Csp_P* ingestion (unpaired t-test, * $p < 0.05$, ** $p < 0.01$). Each replicate included at least 5 mosquitoes, and a total of three biological replicates were conducted.

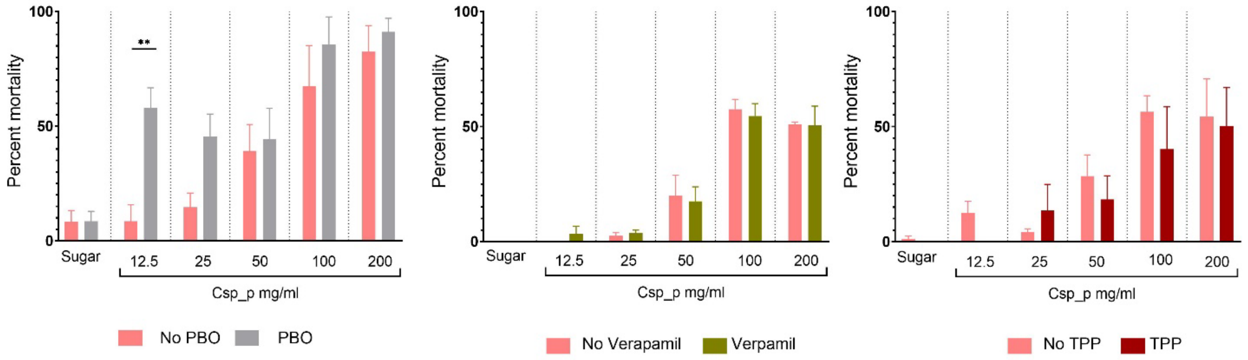

Fig. S3. The impact of pre-exposure to different synergists on *Chromobacterium* sp. Panama (*Csp_P*) induced mortality

3–5-day old *An. gambiae* Keele female mosquitoes were starved for 24 hours and then exposed to either, Piperonyl butoxide (PBO), verapamil, or Triphenyl phosphate (TPP) for one hour in WHO bioassay tubes, followed by feeding on *Csp_P* biopesticide (at concentrations ranging from 12.5 to 200 mg/ml) or a control (10% sucrose) for 24 hours ad-libitum. Mortality was assessed 24 hours after exposure. Pre-exposure to PBO resulted in increased mortality at 12.5 mg/ml of *Csp_P* (Two-way ANOVA, followed by Šídák's multiple comparisons test, ** $p < 0.01$). There was no difference in mortality after pre-exposure to PBO at higher concentrations of *Csp_P* (25, 50, 100, and 200 mg/ml), nor after exposure to verapamil or TPP at any concentration of *Csp_P* tested here (Two-way ANOVA, followed by Šídák's multiple comparisons test, $p > 0.05$). Each replicate included at least 25 mosquitoes, and a total of three biological replicates were conducted.

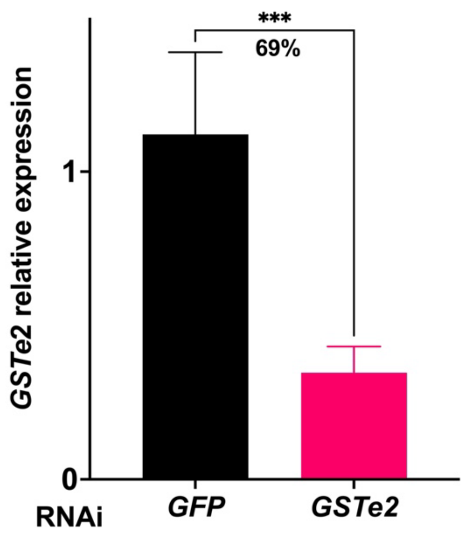

Fig. S4. RNAi-mediated gene silencing efficiency of *GSTe2*

3–5-day old *An. gambiae* Keele female mosquitoes were injected with *GSTe2 dsRNA* or *GFP dsRNA* as control. Relative transcript levels of *GSTe2* were evaluated 72 hours post-injection. There was a significant reduction in the relative expression of *GSTe2* in mosquitoes injected with *GSTe2* dsRNA compared to *GFP dsRNA*-injected control mosquitoes (unpaired t-test, p = 0.0004). Each replicate included at least 5 mosquitoes and a total of four biological replicates were conducted, the bar indicates the mean value, and the error bar (*s.e.*) is included.

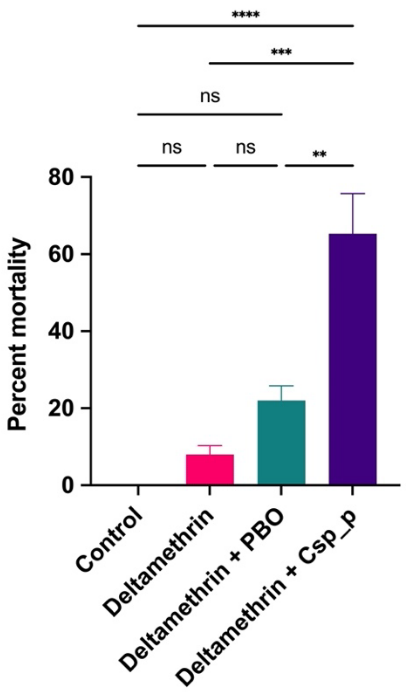

Fig. S5. Synergistic effect of PBO and *Chromobacterium* sp. Panama (*Csp_P*) on deltamethrin in deltamethrin-resistant *An. coluzzii* VK strain

Female mosquitoes of deltamethrin-resistant *An. coluzzii* VK7 strain were pre-exposed to PBO for one hour or were allowed to feed on *Csp_P* (at a non-lethal dose of 50 mg/ml) for 24 hours before being exposed to 0.05% deltamethrin in WHO bioassay tubes for one hour. A solvent control was used for comparison, and mortality was recorded 24 hours post-exposure. Original data for mosquitoes fed on *Csp_P* only is presented in Figure 2C. There was no significant difference in mortality in mosquitoes exposed to 0.05% deltamethrin with or without pre-exposure to PBO. Mosquitoes fed on a non-lethal dose of *Csp_P* exhibited significantly higher mortality compared to all other groups post-deltamethrin exposure (One-way ANOVA p = 0.003, followed by Tukey's multiple comparisons test; *** $p < 0.001$, ** $p < 0.01$). Each replicate included at least 25 mosquitoes, and a total of three biological replicates were conducted, with the bar indicating the mean value along with the error bar (*s.e.*).

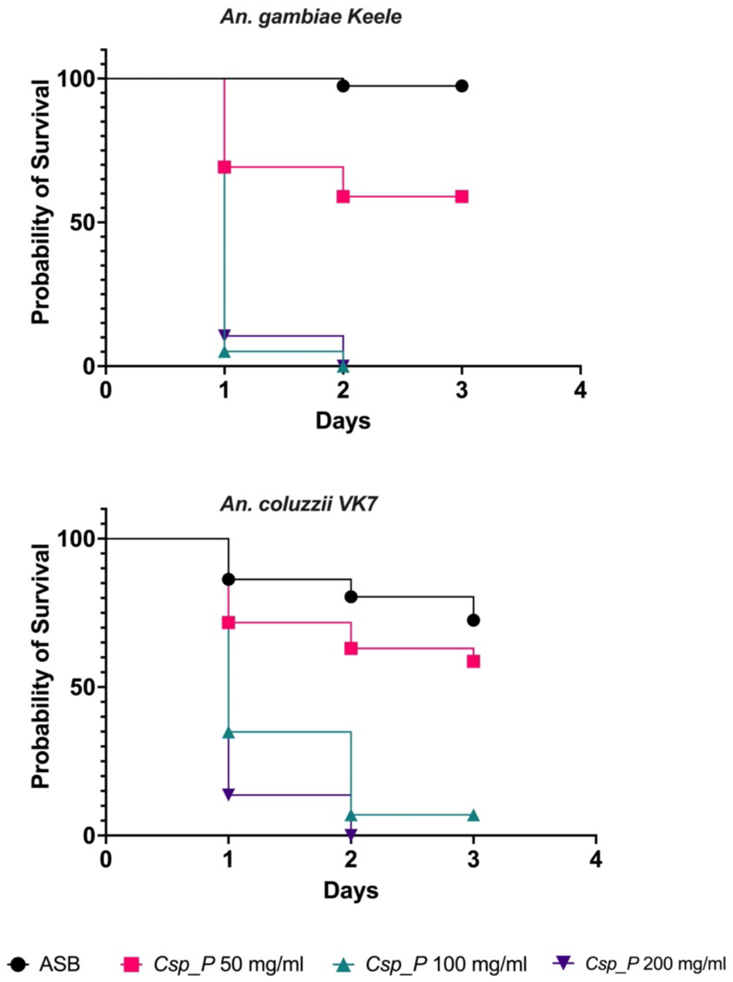

Fig. S6. Mosquitocidal assays with *Chromobacterium* sp. Panama (*Csp_P*) biopesticide combined with an attractive sugar bait (artificial nectar, ASB)

An. gambiae Keele or VK7 female mosquitoes were starved for 24 hours, followed by 24-hour ad-libitum feeding on either 50% artificial nectar (ASB) as a control or a mixture of *Csp_P* at concentrations of 50, 100, or 200 mg/ml mixed with 50% artificial nectar. Results demonstrated that both mosquito strains exhibited lower survival rates when fed on *Csp_P* biopesticide compared to the control at 100 and 200 mg/ml of *Csp_P*, while at 50 mg/ml of *Csp_P* Keele strain exhibited lower survival probability (Log-rank (Mantel-Cox) test, $p < 0.0001$) but VK7 did not ($p = 0.1258$). Each replicate included at least 20 mosquitoes, and a total of three biological replicates were conducted.

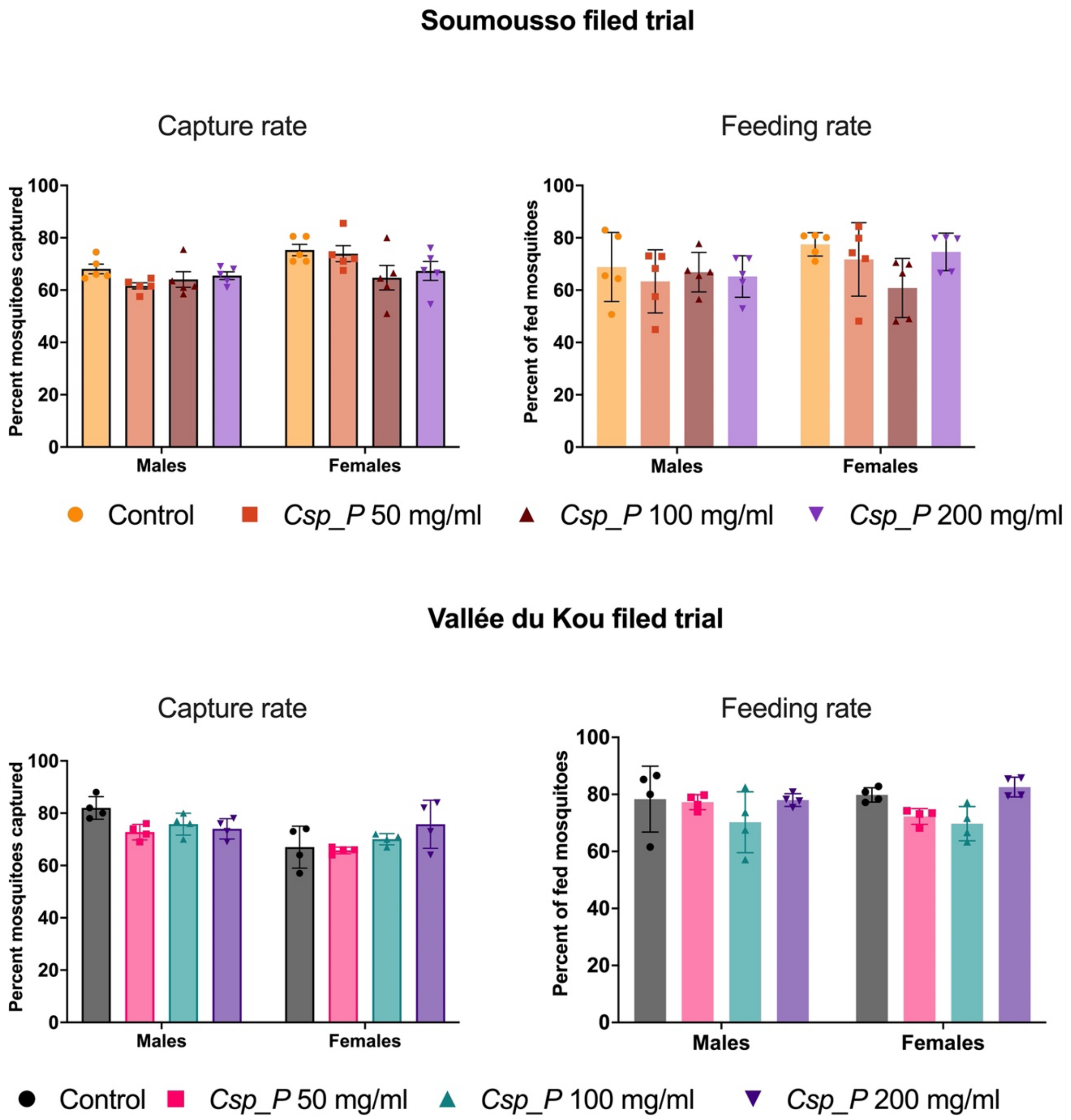

Fig. S7. Capture and feeding rates of mosquitoes from Soumousso and Vallée du Kou enclosed semi-field trials

The capture rate (left) and feeding rate (right) of the mosquitoes after exposure to *Chromobacterium* sp. Panama (*Csp_P*) feeding stations observed in the trials exhibited no significant difference across all treatments for both male and female mosquitoes (Tukey's multiple comparisons test, $p > 0.05$). Each dot on the graph represents values from individual replicates, with bars indicating the mean values along with the error bars (*s.e.*). Each replicate included at least 200 mosquitoes, and a total of four or five biological replicates were conducted.

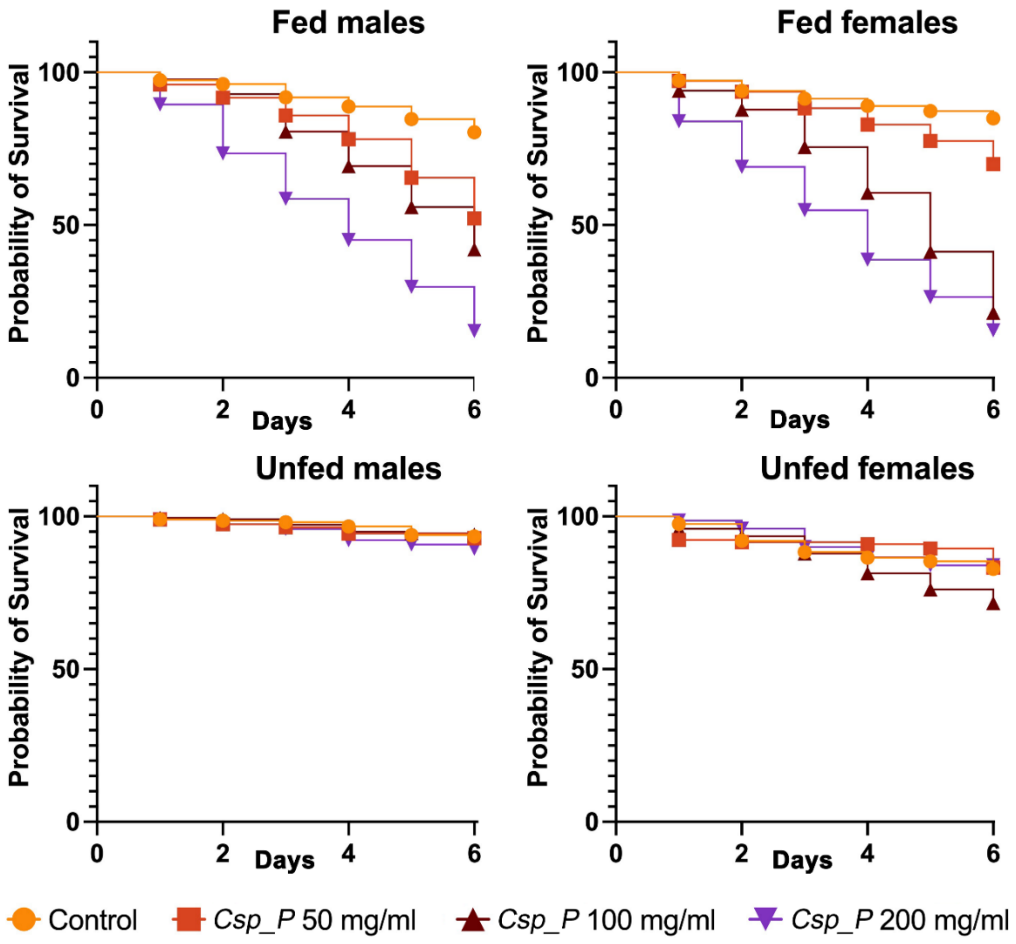

Fig. S8. Mosquitocidal assays at Soumousso enclosed semi-field trials

In the Soumousso field trials, *Chromobacterium* sp. Panama (*Csp_P*) ingestion led to decreased survival probabilities post-ingestion in both males (left top) and females (right top). Specifically, there was significant mortality observed, with 84.51% in males and 84.66% mortality in females by day 6, at the conclusion of the monitoring period (Log-rank (Mantel-Cox) test, $p < 0.0001$). Both unfed males (left bottom) and females (right bottom) showed no difference in survival rates, confirming the mosquitocidal activity of *Csp_P*. Each replicate included at least 200 mosquitoes, and a total of three biological replicates were conducted.

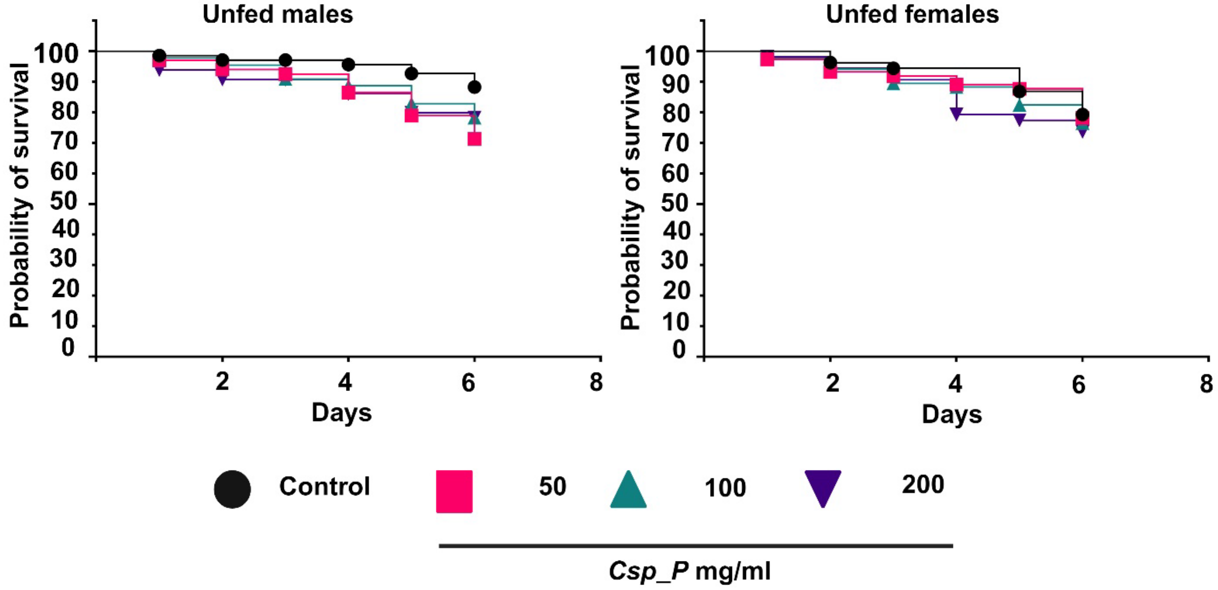

Fig. S9. Survival probabilities of unfed mosquitoes at Vallée du Kou semi-field trials
Survival probabilities of unfed males (A) and females (B) from the Vallée du Kou trials. control (50% artificial nectar) and *Chromobacterium* sp. Panama (*Csp_P* **)** biopesticide (50, 100, 200 mg per ml of 50% artificial nectar). Three independent experiments were performed with 100 female and 100 male mosquitoes in each experiment (Log-rank (Mantel-Cox) test, $p > 0.05$).

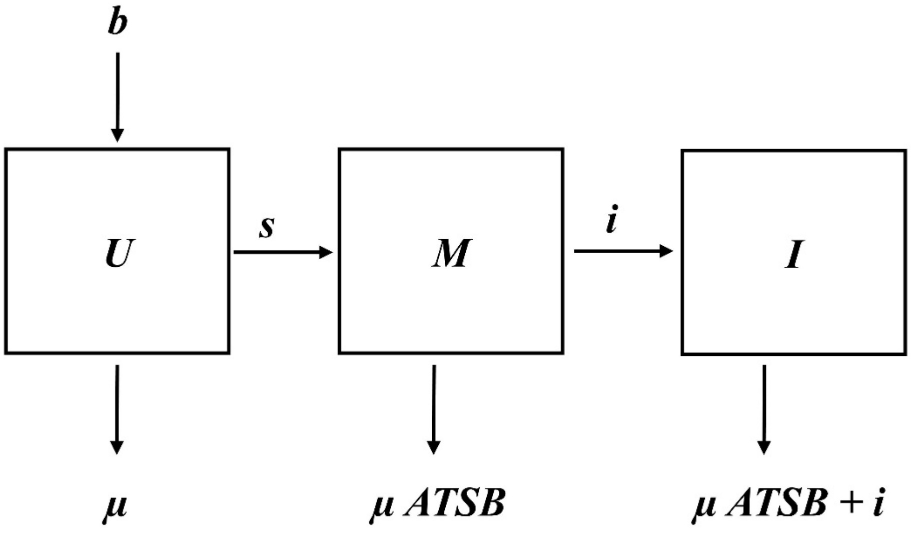

Fig. S10. The modified modeling framework for the impact of the *Chromobacterium* sp. Panama (*Csp_P*) Attractive Toxic Sugar Baits (ATSBs) on mosquito population. Modified model to characterize the dynamics of mosquitoes exposed to *Csp_p* ATSB and insecticide. Here, U, M and I represent the density of mosquitoes unfed, fed, and fed plus exposed to the insecticide, respectively; b is a parameter for the adult emergence rate, s for sugar-feeding rate and μ, μ_{ATSB} and μ_{ATSB+i} are, respectively, the death rates of mosquitoes unfed, fed, and fed plus insecticide exposure.

Supplementary tables

Table S1. Statistical data for Figure 2C, Two-way ANOVA followed by Tukey's multiple comparisons test.

Tukey's multiple comparisons test	95.00% CI of diff.	Summary	Adjusted P Value
An. coluzzii VK			
Control vs. Insecticide	-18.74 to 13.41	ns	0.9709
Control vs. Csp_p	-16.07 to 16.07	ns	>0.9999
Control vs. Insecticide + Csp_p	-76.07 to -43.93	****	<0.0001
Insecticide vs. Csp_p	-13.41 to 18.74	ns	0.9709
Insecticide vs. Insecticide + Csp_p	-73.41 to -41.26	****	<0.0001
Csp_p vs. Insecticide + Csp_p	-76.07 to -43.93	****	<0.0001
An. gambiae VK			
Control vs. Insecticide	-17.41 to 14.74	ns	0.9962
Control vs. Csp_p	-16.07 to 16.07	ns	>0.9999
Control vs. Insecticide + Csp_p	-56.07 to -23.93	****	<0.0001
Insecticide vs. Csp_p	-14.74 to 17.41	ns	0.9962
Insecticide vs. Insecticide + Csp_p	-54.74 to -22.59	****	<0.0001
Csp_p vs. Insecticide + Csp_p	-56.07 to -23.93	****	<0.0001
An. arabiensis RUFISQUE			
Control vs. Insecticide	-17.74 to 14.41	ns	0.9926
Control vs. Csp_p	-28.41 to 3.740	ns	0.1872
Control vs. Insecticide + Csp_p	-81.07 to -48.93	****	<0.0001
Insecticide vs. Csp_p	-26.74 to 5.407	ns	0.302
Insecticide vs. Insecticide + Csp_p	-79.41 to -47.26	****	<0.0001
Csp_p vs. Insecticide + Csp_p	-68.74 to -36.59	****	<0.0001
An. gambiae RSP			
Control vs. Insecticide	-101.1 to -68.93	****	<0.0001
Control vs. Csp_p	-37.74 to -5.593	**	0.0042
Control vs. Insecticide + Csp_p	-114.4 to -82.26	****	<0.0001
Insecticide vs. Csp_p	47.26 to 79.41	****	<0.0001
Insecticide vs. Insecticide + Csp_p	-29.41 to 2.740	ns	0.1357
Csp_p vs. Insecticide + Csp_p	-92.74 to -60.59	****	<0.0001
An.gambiae AKDR			
Control vs. Insecticide	-33.98 to -1.830	*	0.0236
Control vs. Csp_p	-41.31 to -9.160	***	0.0007
Control vs. Insecticide + Csp_p	-109.4 to -77.21	****	<0.0001
Insecticide vs. Csp_p	-23.40 to 8.743	ns	0.6213
Insecticide vs. Insecticide + Csp_p	-91.45 to -59.30	****	<0.0001
Csp_p vs. Insecticide + Csp_p	-84.12 to -51.97	****	<0.0001
An.gambiae ZAN/U			
Control vs. Insecticide	-49.52 to -17.38	****	<0.0001

Control vs. Csp_p	-67.71 to -35.57	****	<0.0001
Control vs. Insecticide + Csp_p	-101.1 to -68.94	****	<0.0001
Insecticide vs. Csp_p	-34.26 to -2.117	*	0.0208
Insecticide vs. Insecticide + Csp_p	-67.64 to -35.49	****	<0.0001
Csp_p vs. Insecticide + Csp_p	-49.45 to -17.30	****	<0.0001

Table S2. Statistical data for Figure 4, Two-way ANOVA followed by Tukey's multiple comparisons test.

Dunnett's multiple comparisons test	95.00% CI of diff.	Summary	Adjusted P Value
Alive-fed		*	0.0343
Control vs. 50 mg/ml Csp_p	31.90	***	0.0007
Control vs. 100 mg/ml Csp_p	81.69	***	0.0008
Control vs. 200 mg/ml Csp_p	86.36		
Dead-fed		ns	0.9764
Control vs. 50 mg/ml Csp_p	0.3814	ns	0.2279
Control vs. 100 mg/ml Csp_p	-2.996	ns	0.6388
Control vs. 200 mg/ml Csp_p	-1.663		
Alive-unfed		ns	0.0522
Control vs. 50 mg/ml Csp_p	-18.96	ns	0.3641
Control vs. 100 mg/ml Csp_p	-1.937	ns	0.3376
Control vs. 200 mg/ml Csp_p	3.104		
Dead-unfed		*	0.0419
Control vs. 50 mg/ml Csp_p	-13.31	***	0.0007
Control vs. 100 mg/ml Csp_p	-76.74	***	0.0001
Control vs. 200 mg/ml Csp_p	-87.79	*	0.0343

table S3. Primers used for gene expression analysis and RNAi-mediated gene silencing and gene silencing validations.

Gene	Accession#	Primer name	Primer sequence (5' → 3')
GFP		dsGFP_F	TAATACGACTCACTATAGGG ATGGTGAGCAAGGGCGAGGAGCTGT
		dsGFP_R	TAATACGACTCACTATAGGG TTGTACAGCTCGTCCATGCCG
GSTe2	AGAP009194	dsGSTe2_F	TAATACGACTCACTATAGGG TTCGGCAAATCGGACATCCC
		dsGSTe2_R	TAATACGACTCACTATAGGG ATCGTTTGCTCTCTGGCACT
GSTe2	AGAP009194	qGSTE2_F	GCCGGAATTTGTGAAGCTAAACCCG
		qGSTE2_R	TGCTTGACGGGGTCTTTCGGAT
RPS7		qRPS7_F	AGAACCAGCAGACCACCATC

RPS7		qRPS7_F	GCTGCAAACTTCGGCTATTC
CYP9K1	AGAP000818	qCYP9K1_F	CCGACACGTGGTGATGGATAC
CYP9K1	AGAP000818	qCYP9K1_R	CGTCGTCGGTCCAGTCAAC
CYP4G16	AGAP001076	qCYP4G16_F	GTCCAAGAAGTTGCGTCGGAC
CYP4G16	AGAP001076	qCYP4G16_R	TCTTCGATTTGCGTTGACGTG
CYP6P4	AGAP002867	qCYP6P4_F	CTGGACAACGTTATCAATGAAACC
CYP6P4	AGAP002867	qCYP6P4_R	GCACGGTGTAATCACGCATC
